# Prevalence of rheumatic valvular heart disease in Rwandan school children: echocardiographic evaluation using the World Heart Federation criteria

**DOI:** 10.5830/CVJA-2017-007

**Published:** 2017

**Authors:** J Mucumbitsi, B Bulwer, L Mutesa, MD, MSc Ndahindwa, M Semakula, E Rusingiza, P Arya, S Breakey, C Patton-Bolman, E L Kaplan

**Affiliations:** Rwandan Heart Foundation and Department of Pediatrics, King Faisal Hospital, Kigali, Rwanda; Noninvasive Cardiovascular Research, Cardiovascular Division, Brigham and Women’s Hospital, Boston, Massachusetts, USA; College of Medicine and Health Sciences, University of Rwanda, Kigali, Rwanda; School of Public Health, College of Medicine and Health Sciences, University of Rwanda, Kigali, Rwanda; Rwanda Biomedical Centre, Kigali, Rwanda; Interuniversity Institute for Biostatistical and Statistical Bioinformatics, University of Hasselt, Belgium; School of Medicine and Pharmacy, University of Rwanda, Kigali, Rwanda; Department of Pediatric Cardiology, Massachusetts General Hospital, Boston, Massachusetts, USA; Massachusetts General Hospital, Institute of Health Professions, Boston, Massachusetts, USA; Massachusetts General Hospital, Institute of Health Professions, Boston, Massachusetts, USA; Department of Pediatrics, University of Minnesota Medical School, Minneapolis, Minnesota, USA

**Keywords:** rheumatic heart disease, rheumatic fever, hocardiography, prevalence, epidemiology

## Abstract

**Background:**

Rheumatic fever (RF) and rheumatic valvular heart disease (RHD) remain important medical, surgical and public health concerns in many parts of the world, especially in sub-Saharan Africa. However, there are no published data from Rwanda. We performed a RHD prevalence study in a randomly selected sample of Rwandan school children using the 2012 World Heart Federation (WHF) criteria.

**Methods:**

Echocardiographic assessment of 2 501 Rwandan school children from 10 schools in the Gasabo district near Kigali was carried out. Resulting data were evaluated by four experienced echocardiographers. Statistical analyses were carried out by statisticians.

**Results:**

RHD prevalence was 6.8/1 000 children examined (95% CI: 4.2/1 000–10.9/1 000). Seventeen met WHF criteria for RHD, 13 fulfilled criteria for ‘borderline’ RHD and four were ‘definite’ RHD. None of these 17 had been previously identified.

**Conclusion:**

These data indicate a significant burden of RHD in Rwanda and support a need for defined public health RF control programmes in children there.

## Introduction

The incidence of acute rheumatic fever (RF) and the prevalence of rheumatic heart disease (RHD) have decreased remarkably in industrialised countries during recent decades.^1^-^3^ Yet this disease process remains a significant health issue among socio-economically disadvantaged populations, especially in developing countries.

To effectively allocate limited health resources for medical and public health planning in low- and middle-income countries, accurate assessment of the current status of RF and RHD is necessary. Echocardiography has emerged as an effective diagnostic tool for assessing cardiac pathology in patients with RHD. Recent studies have reported a wide variation in RF incidence and RHD prevalence across countries, even within different socio-economic strata and geographic regions within the same country.^4^-^7^

Several guidelines for diagnosing RHD have been published in recent years.^8^-^12^ Although some differences exist among these guidelines, they are useful in supporting rapid, non-invasive assessment of RHD status for both clinical care and epidemiological studies. Compared with clinical auscultation, echocardiography has been reported to be advantageous for demonstrating very mild (borderline) pathology or ‘silent carditis/valvulitis’ in RHD.^13^-^15^

The purpose of this project was to determine the prevalence of RHD in a sample of Rwandan school children using the 2012 World Heart Federation (WHF) echocardiographic criteria.^10^ There are no previously published RHD prevalence data from this sub-Saharan African country. Such determinations are important for country-based medical and public health efforts. The findings from our echocardiographic study in Rwanda are described and discussed in this report.

## Methods

Rwanda is a small central African nation of approximately 12 million inhabitants (2013 estimate). The population is relatively young and predominantly rural. The capital city, Kigali, with a population exceeding one million people, is the location of major medical facilities. Other medical facilities such as health centres and hospitals are located at the provincial, district and sector levels.

The Gasabo district, adjacent to the city of Kigali, has a population of approximately 400 000 including its own urban, suburban and rural areas. Data (2011) from the Gasabo District Education Department indicated that there were 106 schools with 67 538 registered primary and 8 989 registered secondary school students.

Rwandan schools are classified by the Rwanda Education Department as rural, peri-urban public and urban private, according to their geographic location and to the socio-economic level of the adjacent population. The majority of schools are in rural areas and are considered ‘economically disadvantaged’. The included population, for the most part, lived in sub-standard housing without running water or electricity.

The sample size calculated for the study was 2 940, assuming a prevalence of definite RHD at 1% in school-aged children (6–16 years), with a precision of 0.4% and an inter-cluster correlation coefficient equal to 0.001. When considering 2% non-respondents, the final sample size was increased to 3 000.

Ten schools from the Gasabo district were selected for this study using a stratified two-stage cluster sampling, where the primary sampling units were schools from the three following areas with different socio-economic levels: rural public schools, middle class and urban private schools.

A second stage of preparation was based on individuals, including all classes in each school where all school children were stratified by grade, class and gender to ensure an equal number of boys and girls from the grades included in the sample. Using official lists of students, children were then randomly selected, using the function RANDBETWEEN (Excel software).

**Fig. 1.**
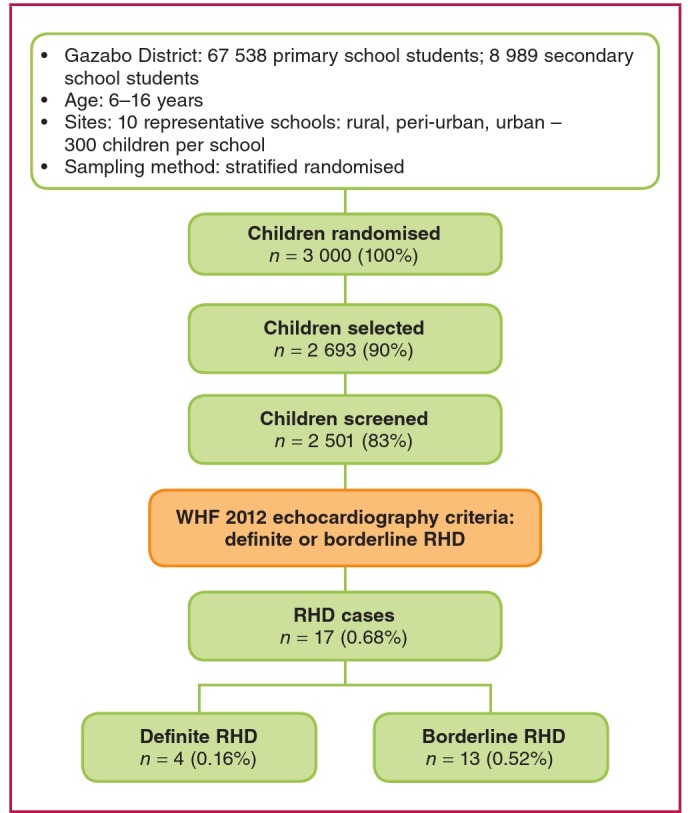
Summary of methodology and echocardiographic screening outcomes of the representative sample of Rwandan school children in the Gazabo district of the Rwandan capital, Kigali, and cases assessed as definite or borderline rheumatic heart disease (RHD) using the WHF 2012 criteria.^9^

The majority of students in five of these 10 schools were classified by the Education Department as being economically disadvantaged. From each of the 10 selected schools, a stratified, randomised selection of 300 students was performed. Random selection of an additional 50 to 100 students from each school was also performed to constitute a reserve list. The original group of 3 000 subjects included those from rural, peri-urban and urban areas of the Gasabo district (Fig. 1).

Informed parental consent, subjects’ assent (if older than eight years), and a questionnaire that included socio-demographic data, as well as personal and family health histories were obtained for each selected subject and alternate subject.

The study protocol was approved by the Rwanda National Ethics Committee and the Rwandan Ministries of Health and of Education. Socio-economic status was classified as high, medium and low according to Gasabo District Education Department criteria.

Educational sessions and materials about RF/RHD and the programme’s objectives were developed and distributed to school teachers, headmasters, administrators and parents of the subjects representing the selected schools. Included were didactic pamphlets, posters and banners in Kinyarwanda, the principal language spoken by most Rwandans.

## Echocardiographic screening procedures

The echocardiographic examinations were performed during a 10-day period by 14 experienced US-certified sonographers who were trained and followed the 2012 WHF criteria for the echocardiographic diagnosis of RHD^10^ (Fig. 1, Table 1). All the sonographers held a certification by either the American Registry of Diagnostic Medical Sonography (ARDMS) or Cardiovascular Credentialing International (CCI). Additionally, the mean number of years of experience exceeded 14 for the sonographers.

**Table 1:** World Heart Federation 2012 criteria for echocardiographic diagnosis of rheumatic heart disease as applied to this study (modified from reference 10).

*Echo criteria for children ≤ 20 years of age*	
Definite RHD (either A, B, C or D):	
A) Pathological MR and at least two morphological features of RHD of the MV	
B) MS mean gradient ≥ to 4 mmHg (NB – exclude congenital MV anomalies)	
D) Borderline disease of both the aortic and mitral valves as defined below Borderline RHD (either A, B or C):	
A) At least two morphological features of RHD of the MV without pathological MR or MS	
B) Pathological MR	
C) Pathological AR	
Normal echocardiographic findings (all A, B and C);	
A) MR that does not meet all four Doppler criteria (physiological MR)	
B) AR that does not meet all four Doppler criteria (physiological AR)	
C) An isolated morphological feature of RHD of the MV or the AV (e.g. valvar thickening) without any associated pathological stenosis or regurgitation
*Echo criteria for adults > 20 years of age*	
Definite RHD (either A, B, C or D):	
A) Pathological MR and at least two morphological features of RHD of the MV	
B) MS mean gradient ≥ to 4 mmHg (NB – exclude congenital MV anomalies)	
C) Pathological AR and at least two morphological features of RHD of the AV in those under 35 years of age only. (Bicuspid AV and dilated aortic root must first be excluded)	
D) Pathological AR and at least two morphological features of RHD of the MV	
Pathological regurgitation	
Mitral regurgitation (all four Doppler criteria must be met)	Aortic regurgitation (all four Doppler criteria must be met)
1. Seen in two views	1. Seen in two views
2. In at least one view jet length ≥ 2 cm	2. In at least one view jet length ≥ 1 cm
3. Peak velocity ≥ 3 m/s	3. Peak velocity ≥ 3 m/s
4. Pansystolic jet for at least one envelope	4. Pandiastolic jet for at least one envelope
Morphological features of RHD	
Mitral valve	Aortic valve
1. AMVL thickening ≥ 3 mm (age-specific)	1. Irregular or focal thickening
2. Chordal thickening	2. Coaptation defect
3. Restricted motion	3. Restricted motion
4. Excessive leaflet tip motion during systole (hypermobile or flail leaflet) resulting in abnormal coaptation	4. Prolapse

RHD, rheumatic heart disease; MR, mitral regurgitation; MV, mitral valve; MS, mitral stenosis; AR, aortic regurgitation; AV, aortic valve; AMVL, anterior mitral valve leaflet.

The echocardiographic instruments, along with appropriatesized multi-Hertz, phased-array transducers, included: 11 SonoSite (five Micromaxx + five Turbo + one Nanomaxx – all SonoSite Inc, Bothell, Washington), one Philips CX50 (Philips Ultrasound, Bothell, Washington), one Acuson Cypress (Siemens, Mountain View, California) and one GE Vivid i (GE Medical Systems, Milwaukee, Wisconsin).

Transthoracic echocardiographic examinations were conducted in specially prepared rooms at each school. The following views were obtained from each subject: parasternal long-axis view, parasternal short-axis views, apical three- and four-chamber views, along with colour-flow Doppler and spectral Doppler interrogation of the intracardiac valves.

Two paediatric cardiologists were present during the echocardiographic screening examinations. During or following each echocardiographic examination, the sonographer and paediatric cardiologist preliminarily discussed any findings of suspected significance. Concomitant physical examinations, including cardiac auscultation were not performed.

The completed studies were uploaded to a cloud-based web server and stored using a picture archiving and communication system (PACS) (Studycast; Core Sound Imaging, Inc, Raleigh, North Carolina) and proprietary software (CoreConnect; Core Sound Imaging, Inc). The transfer of the echocardiographicimages and data occurred via a secure broadband internet connection, with CoreWeb validating the integrity and confidentiality of the transmitted studies using standard secure (SSL, TLS) encryption.

## Post-screening echocardiographic analysis

Two certified professionals initially viewed each echocardiogram. These included an echocardiographer and a paediatric cardiologist with level III training in echocardiography (American Society of Echocardiography), who separately interpreted and reported on each archived echocardiographic examination using a standardised format (SmartWorksheetsTM, CoreWeb, North Carolina). During this phase of analysis the readers were blinded to individual demographic data.

The echocardiographic descriptions focused on findings summarised in the WHF 2012 criteria for RHD and were categorised as definite RHD, borderline RHD, or no RHD (Table 1).^10^

Physiological (non-pathological) tricuspid valve and pulmonary valve regurgitation were noted, but not comprehensively assessed unless there was significant co-existing aortic and/or mitral valve pathology

Subjects who were suspected of having echocardiographic features of RHD or congenital heart disease were then referred and examined by the Kigali-based paediatric cardiologists (JM, ER) who are experienced in the diagnosis and management of RHD, for subsequent management.

## Statistical analyses

Data management and statistical analyses of all data were performed (LM, VN, MS) at the Rwanda Biomedical Centre in the Medical Research Centre in Kigali using STATA software (Statacorp LP. College Station, Texas).

Descriptive statistics were performed; data were summarised using frequency tables and graphs. Confidence interval of RHD prevalence was computed at the 95% confidence level.

## Results

Of the original 3 000 school children randomly selected, 2 693 (89.7%) underwent echocardiographic evaluation. However on their scheduled day for screening, 307 were not present at school or were not available. Of these 2 693 subjects, complete demographic data from 2 501 subjects were available (92.8%; this is 83.3% of the original 3 000 selected subjects) (Fig. 1).

**Fig. 2.**
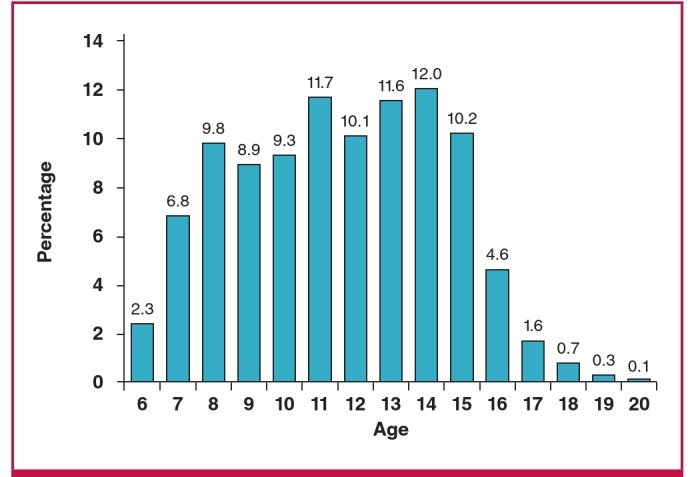
Age distribution of the 2 501 Rwandan school children studied echocardiographically.

The age distribution of the 2 501 subjects is shown in Fig. 2. The mean age of these 2 501 subjects who were completely analysed was 11.2 years.

Of importance, 91% of the subjects undergoing diagnostic echocardiography were students enrolled in the first six years of primary school. Approximately 9% of those echocardiographically evaluated were somewhat older students (ages 16–20 years) enrolled in the first three years of secondary school. Therefore, the overall age distribution for the 2 501 subjects was weighted towards younger school children.

Seventeen of the 2 501 children (0.68%) fulfilled the 2012 WHF echocardiographic criteria for the diagnosis of RHD, in either the definite or borderline category (Table 2, modified from reference 10). Therefore, the RHD prevalence among this sample of 2 501 Rwandan school children was 6.8/1 000 (95% CI: 4.2/1 000–10.9/1 000).

**Table 2:** Summary of echocardiographic findings among the 17 subjects found to have rheumatic valvular heart disease

*WHF category*	*Valve lesion*	*Male*	*Female*	*Total*	*Percentage*
Subjects with RHD using WHF criteria	17	13	4	17	0.68
Borderline	Pathological MR	10	3	13	0.52
	Pathological AR	0	0		
Definite	Pathological MR	2	1	4	0.16
	Pathological AR	1	0		

For complete 2012 WHF diagnostic criteria in children (and adults), refer to reference 10.

## Characteristics of subjects meeting WHF criteria for RHD

**Fig. 3.**
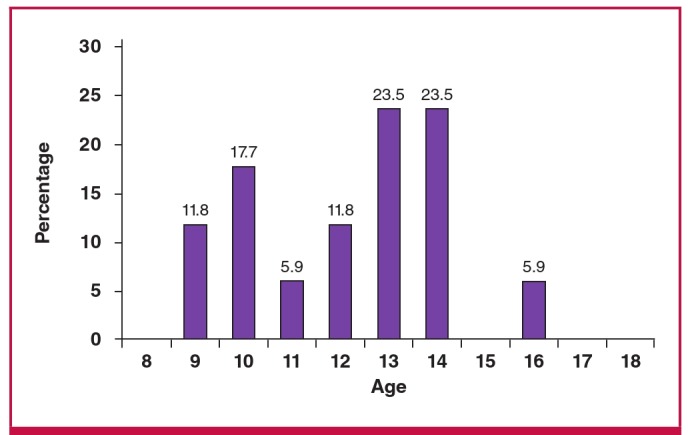
Age distribution of the 17 subjects diagnosed with rheumatic valvar heart disease using the WHF 2012 echocardiographic criteria.^9^

**Fig. 4.**
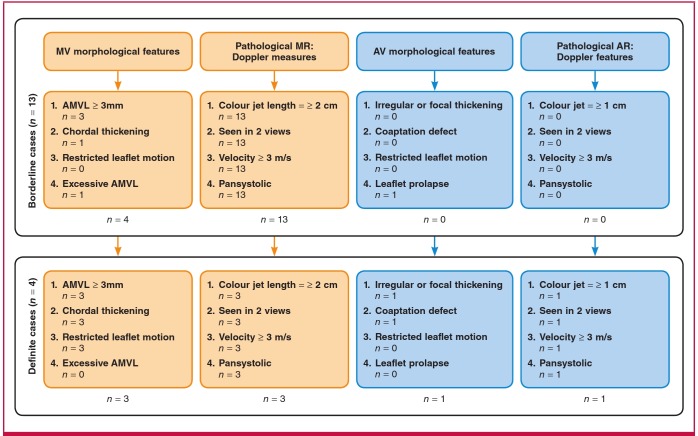
Children assessed as definite or borderline RHD using the morphological and Doppler criteria for RHD according to the WHF 2012 criteria.^9^

The age distribution of the 17 subjects with valvular RHD is shown in Fig. 3. Of the 17 subjects who met the 2012 WHF criteria, four (23.5%) were identified as definite RHD and 13 (76.5%) subjects were classified as borderline RHD (Fig. 4).

The mean age of the 17 subjects found to have RHD was 12.2 years, which was slightly greater than the mean age (11.2 years) of the 2 501 evaluated group. This discrepancy in ages was not statistically significant. In fact, among the group of previously described older children, there was also a higher prevalence of RHD when compared with the younger age group. However, these age differences were not statistically significant (p = 0.856).

Mean weight and height for these 17 subjects were 35.68 kg (78.5 lbs) and 139.88 cm (55.1 inches), respectively. Forty-nine per cent were male and 51% were female. While there was a higher prevalence of echocardiographic valvular findings in the males when compared with female subjects, this difference was not statistically significant (p = 0.062).

The individual echocardiographic findings for the 17 subjects who fulfilled the WHF criteria are depicted in Fig. 4. As would be expected, the most frequently affected valve was the mitral valve. There was only one subject with solitary aortic valve findings.

**Fig. 5.**
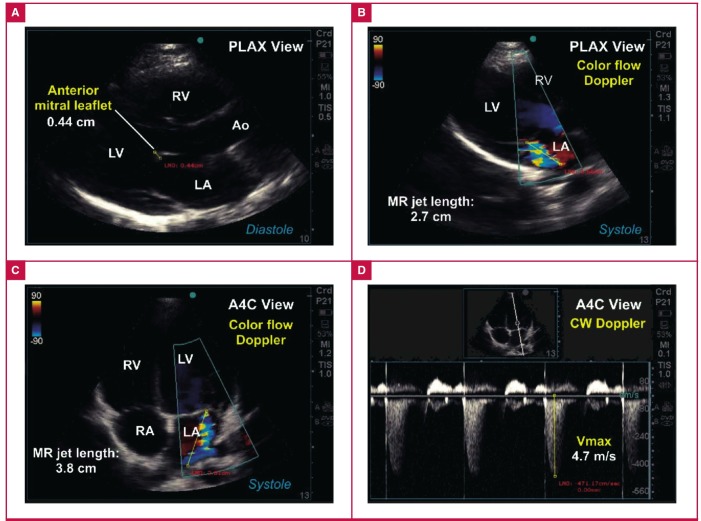
Example of definite RHD consistent with WHF 2012 criteria. A. Parasternal long-axis (PLAX) view showing thickened anterior mitral leaflet exceeding 3 mm in young adult study subject. B. Colour-flow Doppler interrogation of the mitral valve shows an eccentric jet of mitral regurgitation (MR) with jet length exceeding 2 cm. C. Colour-flow Doppler interrogation of the mitral valve using the apical four-chamber (A4C) view shows eccentric jet of mitral regurgitation with jet length measuring 3.8 cm. D. Continuous-wave (CW) Doppler interrogation of the MR jet showed maximum velocities exceeding 4.7 m/s.

**Fig. 6.**
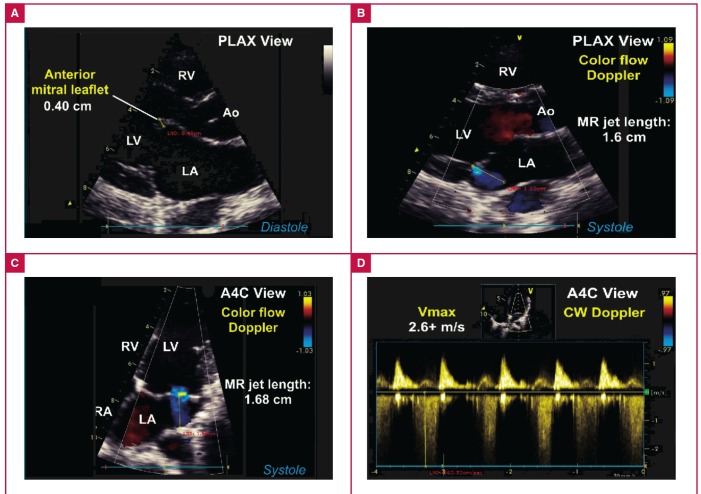
Example of borderline RHD consistent with WHF 2012 criteria. A. Parasternal long-axis (PLAX) view showing thickened anterior mitral leaflet exceeding 3 mm and restricted leaflet motion in young adult study subject. B. Colour-flow Doppler interrogation of the mitral valve in the PLAX view shows a small jet of mitral regurgitation (MR) with jet length < 2 cm. C. Colour-flow Doppler interrogation of the mitral valve using the A4C view shows jet of mitral regurgitation with jet length < 2 cm. D. Continuous-wave (CW) Doppler interrogation of the MR jet showed maximum velocities exceeding < 3 m/s.

An example of definite valvular RHD (subject #06-0206) is illustrated in Fig. 5. By contrast, an example of borderline RHD (subject #06-0168) is shown in Fig. 6.

The majority of the 17 subjects (76%) echocardiographically classified as having RHD were categorised as ‘economically disadvantaged students’. Additionally, 13 of the 17 subjects (76%) with echocardiographic findings compatible with RHD were students at schools that were demographically classified as rural. Although not of statistical significance, these trends are important and may have significant medical care and public health programme implications.

Of the 2 501 subjects completely analysed, only two (0.03%) were found to have congenital heart disease (a 2.8-cm secundum atrial septal defect and a small pressure-restrictive patent ductus arteriosus). No other significant underlying chronic medical problems were identified among the subjects during these cardiac-specific evaluations. No subjects demonstrated heart failure and no subjects with acute rheumatic fever were identified.

## Discussion

RHD prevalence reports from echocardiographic screening studies in sub-Saharan Africa vary and high prevalence rates are not uncommon.^6^,^7^,^13^-^15^ To date, no published data exist about RHD prevalence rates among school children in urban or rural Rwanda. The geographic district of Gasabo was identified because it was socio-economically diverse, as well as being near Kigali city. Because of this, and following consultations with the Republic of Rwanda Ministry of Health and school authorities, and approval by the Rwandan National Ethics Committee, this project was initiated.

If this reported sample, randomly selected from the approximately 67 000 school children in the Gasabo district, is representative, the results (the prevalence of valvular RHD was determined echocardiographically to be 6.8/1 000), suggest there could be more than 500 school children with established RHD in this single Rwandan district alone. Assuming a constant prevalence across the country (which may be be unjustified) extrapolating this RHD prevalence rate to Rwanda’s total population of school children could prove worrisome with regard to the accompanying medical, public health and economic impacts for the country. Such an extrapolation would most likely indicate that the majority of Rwandan school children with RHD are most likely undiagnosed and not receiving recommended medical care, including secondary antibiotic prophylaxis. Our data further suggest that the preponderance of RHD is most likely in rural Rwanda. Anticipated studies in additional rural areas of the country would clarify this.

In extrapolating the present Gasabo data for future public health planning, the probability of higher RHD prevalence rates among adolescents and young adults must also be confirmed. If true, this would require extended coverage by public health RHD control programmes and medical facilities beyond the primary school-age population.

The echocardiograms analysed here were obtained by very experienced, registered cardiac sonographers with an average of 14 years of experience and were aditionally analysed by experienced echocardiographers (JM, BB, PA, ER) who had been given access to an advanced draft of the 2012 WHF criteria and were aware of the 2012 criteria.

The RHD school-age prevalence of approximately seven per 1 000 children from this single Rwandan district is lower than, for example, recent reports from Mozambique, Kenya, Uganda, Ethiopia or South Africa.^7^,^13^-^15^ The reasons for these differences are not obvious although variations in prevalence rates were seen in the previously reported studies. While all of these had high prevalence rates and the prevalence rate reported here was lower, one cannot assume that prevalence rates across sub-Saharan Africa are similar. For example, this finding may be related to the fact that our study was carried out in and around the capital city, which may have benefited from better access to healthcare during the last 10 or 15 years, compared with the rest of the country. Additionally, Rwanda has been shown to have invested in primary healthcare and achieved among the best health indicators in sub-Saharan Africa.^16^

There appeared to be a relationship (although not statistically significant, p = 0.704) between the estimated socio-economic status and prevalence of RHD in this sample of Rwandan school children. Engle et al. have shown similar findings with a significantly higher RHD prevalence rate in a more socioeconomically disadvantaged community in South Africa.^17^

The prevalence of RHD in the small group of older school children/adolescents was higher than in the younger school children. With valvular damage becoming more evident with elapsed time,the cumulative prevalence of RHD would be expected to increase with progressing age among children and adolescents. Of note is the fact that in the report by Engle and colleagues, the population appeared somewhat older than in our present population. This may have influenced the higher prevalence reported by these workers.

Increasing age would also offer more opportunities for recurrences of RF, since it appears that none of these children had been previously diagnosed and therefore were not receiving prophylactic antibiotics.

In those 17 subjects found to have either ‘definite’ (n = 4) or ‘borderline’ (n = 13) valvular RHD, the mitral valve was most often affected (Table 2). Again, none of those subjects who met WHF criteria for RHD had been previously diagnosed. Therefore, none of the 17 subjects had been receiving secondary RF prophylaxis prior to this project. In fact, we were unable to elicit a history of RF from any of the 2 501 echocardiographically examined subjects as it has been found in other somewhat similar studies.7 This observation requires additional investigation.

An important challenge facing the application of the WHF 2012 criteria pertains to its specificity in our presumed higher-risk Rwandan population. Evidence from a recent study in a low-risk population suggests high specificity of the morphological mitral and aortic valve features of RHD, but low specificity for the isolated pathological mitral regurgitation criteria.^17^ Of note, almost 95% of our borderline RHD cases were based on the mitral regurgitation criteria. Therefore the issue of specificity of the morphological and Doppler-assessed valvular regurgitation criteria is an important one, and appropriate follow up of such identified patients is mandatory.

The WHF 2012 criteria used during this evaluation^10^ differed significantly from earlier criteria used in several other published studies conducted in the sub-Saharan region. Those studies employed earlier clinical and/or echocardiographic criteria for diagnosing valvular RHD.^13^-^15^ The present report is the second that used only the 2012 WHF echocardiographic criteria in a sub-Saharan school-age population.^7^

We believe that our assessment of these 2 501 subjects accurately adhered to the published WHF criteria. However, we recognise that the final conclusion will depend on long-term (prospective) follow up, which should include not only subsequent serial echocardiograms, but also documentation of adherence to secondary antibiotic prophylaxis for those subjects who initially met the criteria for valvular RHD.^18^ Secondary prophylaxis would help to reduce/eliminate the possibility of recurrent attacks of RF, which would confuse long-term follow-up studies.

The 2015 revised Jones Criteria for the Diagnosis of ARF from the American Heart Association have been endorsed by the World Heart Federation.^12^ Both guidelines underscore the key advantages of using Doppler echocardiography in the diagnosis of ‘subclinical carditis’ – an important diagnostic criterion.

## Limitations

Questions may arise about whether the selected subject sample was representative of all similar-age school children in Rwanda. Students in the Gasabo district were deemed by local authorities and the investigators to be representative of the socio-demographics of school children in Rwanda. Selecting a district closer to the capital was a practical consideration, but could possibly have introduced bias.

Second, the smaller percentage (9%) of relatively older children in the examined sample may have (unintentionally) affected the calculated overall prevalence of RHD, as has been previously mentioned.

Third, technical aspects of the echocardiographic studies require consideration. Although care was taken to standardise both equipment settings and transducer selection in an effort to minimise technical error, we used equipment from different manufacturers. The same paediatric transducers were not universally used for all subjects. Our care to include only experienced, registered sonographers as well as subsequent independent reporting by four experienced US-certified cardiologists/echocardiographers reflects the intent to minimise such potential sources of error.

The absence of echocardiographic findings compatible with congenital bicuspid aortic valves (BAV) in the present study was an unexpected finding. Congenital BAV is a frequent differential when considering rheumatic aortic valvular disease. On average, approximately 1% of children have been reported to have congenital BAV. The reason(s) for the unexpectedly low prevalence of BAV among these Rwandan school children is unclear and requires confirmation.

## Conclusions

These data from a single geographic district of Rwanda confirm the significant prevalence of RHD, as has been reported from other sub-Saharan African countries.^13^-^15^ The feasibility of echocardiographic screening of relatively large numbers of subjects using the 2012 WHF criteria for detecting mild/‘borderline’ RHD in these populations appears to be confirmed. However, intensive and prospective long-term (years) follow up is required to support the conclusions from this sample. The present data will prove useful to health authorities in determining resource planning and allocation for control programmes for this preventable cardiovascular disease.

RF and RHD remain important medical and public health issues in sub-Saharan Africa. Cost-effective public health control programmes are urgently needed.^16^,^17^ Educational efforts targeting healthcare professionals, lay populations (including school children) and public health authorities must be included. The data presented here, while informative, need to be further supported by additional comprehensive studies, including sustainable, cost-effective approaches involving less-expensive and highly portable echocardiographic instruments as well as larger study samples.
